# Enhanced antifouling and antibacterial performances of novel UV-curable polysiloxane/microcapsules/Ag composite coatings for marine applications

**DOI:** 10.1098/rsos.240090

**Published:** 2024-06-05

**Authors:** Ze Liu, Nan Zheng, Jie Liu, Bo Jia, Xiaojun Wang, Pan Yao, Yayu Zhang, Fu Xia, Xinyu Guo

**Affiliations:** ^1^ Shaanxi Key Laboratory of Catalysis, School of Chemical and Environmental Sciences, Shaanxi University of Technology, Hanzhong, Shaanxi 723001, People's Republic of China

**Keywords:** microcapsules, low-surface energy, antifouling coatings, bionics, antibacterial performance

## Abstract

Marine biological fouling is a widespread phenomenon encountered by various oceanic ships and naval vessels, resulting in enormous economic losses. Herein, novel 4,5-dichloro-2-octyl-isothiazolone@sodium alginate/chitosan microcapsules (DCOIT@ALG/CS) were prepared through composite gel method using DCOIT as core materials, ALG and CS as shells, and CaCl_2_ as the cross-linking agent. The formed microcapsules (MCs) with Ag nanoparticles (AgNPs) were then filled in UV-curable polysiloxane (UV-PDMS), followed by UV irradiation to yield UV-PDMS/microcapsules/AgNPs (UV-PDMS/MCs/Ag) composite coatings. The constructed micro–nano dual-scale surface using the MCs and AgNPs improved the antifouling and antibacterial properties of UV-PDMS/MCs/Ag coatings. The as-obtained UV-PDMS/MCs/Ag coatings exhibited a static contact angle of about 160°, shear strength of 2.24 MPa, tensile strength of 3.32 MPa and elongation at break of 212%. The synergistic bacteriostatic effects of DCOIT and AgNPs in UV-PDMS/MCs/Ag coatings resulted in a bactericidal rate of 200 μg ml^−1^ towards *Escherichia coli* and *Staphylococcus aureus* with saturation at 100% within 10 min. In sum, the proposed composite coatings look promising for future marine transportation, pipeline networks and undersea facilities.

## 1. Introduction

Marine fouling is a key technical challenge in marine transportation and underwater facilities requiring urgent solutions [1,2]. Marine fouling refers to the colonization and settlement of microorganisms, plants, algae or small animals on surfaces of undersea substrates, seriously affecting the normal operation of ships and undersea facilities [3]. Nearly 90% of global trade is carried out through the shipping industry [4] but biofouling results in billions of dollars in damage to the shipping industry annually [5].

Microcapsules (MCs) are very effective micron-sized containers for delivering active ingredients, protecting not only the active ingredient from harsh environments but also prolonging the release of encapsulated ingredients [6,7]. As a result, microencapsulation technology [8,9] has attracted great attention in biology [10], medicine [11], textiles [12], food [13], cosmetics [14] and other fields. More importantly, the sustained release effect of MCs [15] has attracted increasing interest in antibacterial [16] and marine antifouling [17].

Sodium alginate (ALG) [18] and chitosan (CS) [19] have widely been employed to prepare MCs as wall materials due to their advantageous biological properties [20], such as non-toxicity, good biodegradability and high biocompatibility. ALG is an anionic linear polysaccharide containing 1,4-linked D-mannuronic acid and L-guluronic acid residues. On the other hand, CS abundantly present in shells of crustaceans and exoskeletons of insects, is a cationic polysaccharide derived from chitin. Thus, simultaneous electrostatic interaction between protonated amino groups of CS and carboxyl groups of ALG forms polyelectrolyte complex MC membranes [21]. Such polyelectrolyte membranes effectively prevent the external environment from damaging the MCs [22].

A typical example of a broad-spectrum antifouling agent consists of 4,5-dichloro-2-octyl-3-isothiazolinone (DCOIT), characterized by high efficiency, low toxicity, long-lasting efficacy and environmental friendliness [23,24]. DCOIT is biodegradable with a half-life in natural seawater of 24 h to 3 days [25]. As a result, DCOIT has broad application prospects in the antifouling industry. DCOIT can easily cross cell membranes and cell walls, resulting in oxidative stress in the cell, and eventually cell necrosis [26]. The toxicity mechanism of DCOIT consists of the formation of free radicals followed by the blockage of the oxidative stress defence system. Besides, DCOIT inhibits glutathione reductase by irreversibly binding to the active centre of the enzyme, thereby reducing the amount of glutathione in the cell [27]. Therefore, DCOIT has been approved in the EU as an active substance in biocidal products of type 21—antifouling agents [28].

The three widely used types of marine antifouling materials consist of low-surface-energy materials, bionic surface materials [29] and release materials. However, these materials suffer from several drawbacks. For instance, these low-surface-energy materials have poor adhesion and mechanical properties, making them prone to easily fall off from the substrate when subjected to water flow [30]. By comparison, biomimetic surface materials have shown better antifouling performance [31] but lack a standard micro-/nano-structural configuration to reduce the settlement of marine organisms. On the other hand, these materials are expensive and difficult to apply universally [32]. Release materials contain toxic antifouling agents, such as tributyltin, which are problematic for damaging the marine environment [33]. Worse, the short release period of the antifouling agents results in a difficult steady and slow-release process [34]. Therefore, the design and preparation of novel environmentally friendly materials combining with excellent mechanical, antifouling, antibacterial properties and long service life are of great research significance.

Accordingly, a multi-combined antifouling strategy was developed using DCOIT as core materials and ALG/CS as shells to prepare DCOIT@ALG/CS MCs by composite gel method, to achieve controlled release of the core material encapsulated inside the shell. The obtained MCs showed a stronger antibacterial effect and longer antibacterial time. The as-obtained MCs and Ag nanoparticles (AgNPs) were then used to construct micro–nano dual-scale biomimetic structures on the surface of low-surface-energy polysiloxane coatings to enhance the antifouling and bacteriostatic properties of the UV-curable polysiloxane/microcapsules/AgNPs (UV-PDMS/MCs/Ag) coatings, as well as extend the service life of the coatings. The UV-PDMS/MCs/Ag coatings exhibited superior mechanical, antifouling and antimicrobial properties based on a series of test results, which were expected to be widely used in marine transportation, pipeline networks and undersea facilities.

## 2. Material and methods

### 2.1. Materials

The raw materials glycidyl methacrylate (GMA, 96%), 1,1-diphenylethene (DPE, 98%), calcium chloride (CaCl_2_), polyvinylpyrrolidone (PVP), pentaerythritol tetra-3-mercaptopropionate (PETMP, 95%), 1H,1H,2H,2H-perfluorodecyltriethoxysilane (PFDS, 96%) and *N*,*N*-dimethylformamide (DMF, 99%) were all purchased from Shanghai Macklin Biochemical Co., Ltd. (Shanghai, China). DCOIT (98%) was supplied by Shanghai Energy Chemical Co., Ltd. (Shanghai, China). ALG (99%), CS (99%) and potassium persulphate (KPS, 99%) were provided by Sinopharm Chemical Reagent Co., Ltd. (Shanghai, China). Sodium hydroxide (NaOH), ethylene glycol (EG, 98%) and acetic acid (CH_3_COOH, 99%) were obtained from Tianjin Kermel Chemical Reagent Co., Ltd. (Tianjin, China). Silver nitrate (AgNO_3_) was purchased from Shanghai Fine Chemical Materials Research Institute (Shanghai, China). Polyvinyl silicone oil was produced by Fujian Polymerization New Material Co., Ltd. (Fujian, China). Dibutyltin dilaurate (DBTDL, 95%) and ethyl-2,4,6-trimethylbenzoylphosphonate (TPO, 97%) were provided by Shanghai Aladdin Biochemical Technology Co., Ltd. (Shanghai, China). Yeast dip powder and agar powder were supplied by Beijing Aoboxing Biology Technology Co., Ltd. (Beijing, China). Polyglycidyl methacrylate (PGMA) was made in laboratory.

### 2.2. Preparation of PGMA

The process consisted of adding 21 g of GMA, 0.27 g of DPE and 240 ml of water into a 500 ml three-necked round-bottomed flask heated to 80℃ under 200 r.p.m. stirring. Subsequently, 20 ml of KPS solution (0.2 g KPS) was added to the above mixture. The polymerization process was then initiated and lasted for 16 h under the protection of a nitrogen atmosphere. After completion of the reaction and temperature drop to 55℃, 600 μl of concentrated sulphuric acid was added, and the reaction was left to react for another 12 h to yield a PGMA solution.

### 2.3. Preparation of MCs

**Figure 1.**
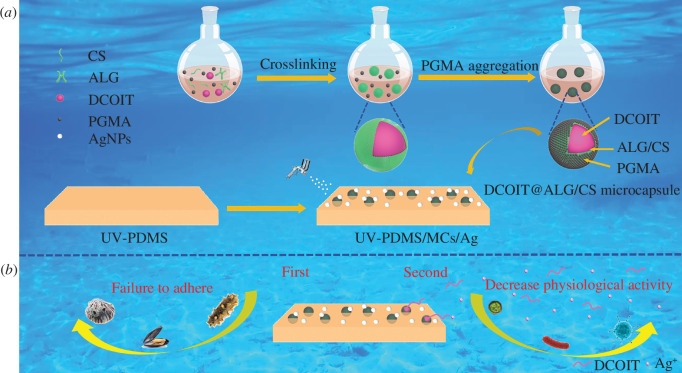
(*a*) Preparation route of MCs and UV-PDMS/MCs/Ag coatings, (*b*) antifouling mechanism of UV-PDMS/MCs/Ag coatings.

The preparation route consisted of mixing 3.92 g of PGMA and 1.275 g of DCOIT in a 50 ml triple-necked flask under stirring at 35℃ and 600 r.p.m. for 5 min. Next, 0.0150 g of ALG was completely dissolved in 1.7 ml of distilled water, and the ALG solution was added to the triple-necked flask under stirring at 2400 r.p.m. for 10 min. Finally, 0.1125 g of CS was completely dissolved in 5 ml of 1.0 vol% acetic acid, and the CS solution was added to the triple-necked flask under stirring at 600 r.p.m. for 10 min. The pH of the solution was adjusted to 6 by adding 5 wt% NaOH under stirring for 10 min. The reaction was then heated to 80℃, followed by a drop-by-drop addition of 5 ml of 0.6 w/v% CaCl_2_ solution. The reaction was carried out for 4 h and the resulting product was washed three times by centrifugation at 3000 relative centrifugal force (RCF) in distilled water followed by freeze drying to obtain MCs. The preparation route of MCs is shown in figure 1*a*. The preparation process of the shells was the same as the aforementioned method, except that the core material DCOIT has not been added.

### 2.4. Preparation of AgNPs

The procedure consisted of adding 280 ml of EG and 8 g of PVP to a three-necked flask under ultrasonic treatment. Next, 0.2 g of AgNO_3_ was dissolved in 20 ml of EG under ultrasonic treatment to yield an AgNO_3_ solution with a light red colour. Afterwards, the obtained AgNO_3_ solution was added drop-by-drop under ultrasonic. During the ultrasonic process, the water temperature was kept at 50℃. The three-necked flask was then placed in an oil bath and the temperature was slowly increased to 140℃ at the rate of 4℃ min^−1^, then keep stirring at 140℃ for 22 h. Finally, the flask was taken out of the oil bath and cooled down naturally to room temperature. The obtained product was washed three times with DMF at 11 000 RCF followed by distilled water twice, and finally ethanol twice for 20 min each time. The resulting product was dried in a blast drying oven at 70℃, and the formed AgNPs were collected. The obtained AgNPs powder was stored in a sealed glass bottle with nitrogen atmosphere to prevent oxidation.

### 2.5. Preparation of UV-PDMS/MCs/Ag coatings

The preparation route consisted of evenly mixing 2 g of polyvinyl silicone oil, 0.1 g of PETMP, 0.04 g of DBTDL, 0.1 g of AgNPs and 0.1 g of MCs, followed by vacuum drying and reaction at 25℃ for 24 h. Next, 0.04 g of TPO was added and stirred uniformly. The resulting mixture was coated on different substrates (glass, ceramics, film, cotton and steel), then 0.1 g of AgNPs, 2 g of acetone and 0.05 g of PFDS were stirred evenly and sprayed onto the surface of the aforementioned mixture. Finally, cured under a UV-LED lamp for 3 min to yield UV-PDMS/MCs/Ag coatings. The UV-PDMS coatings were obtained by the same method but without adding MCs and AgNPs. The preparation route of the UV-PDMS/MCs/Ag coatings is summarized in figure 1*a*.

### 2.6. Characterization

The morphologies and sizes of the MCs were viewed by optical microscopy (OM; Shanghai Caikon Optical Instrument Co., Ltd., Shanghai, China) according to DMM-300C. The size distribution of MCs and AgNPs was measured by a laser particle analyser DLS LS13320 (Beckman Coulter Instruments, Brea, CA, USA). Fourier transform infrared (FT-IR) spectroscopy tests were recorded on VERTEX70 spectroscopy (Bruker, Germany). The test samples were prepared by grinding the MCs with potassium bromide, followed by scanning in the infrared region. Nuclear magnetic resonance spectra (NMR) was measured by ADVANCE III HD 600MHz (Bruker, Munich, Germany). The X-ray diffraction pattern (XRD) was measured by D8 ADVANCE X-ray diffractometer (Bruker). The absorbance spectra of the solutions were tested by Cary 5000 UV–Vis–NIR UV spectrophotometer (Agilent Technologies, Santa Clara, CA, USA). The shear strengths, tensile strengths and elongations at break of the UV-PDMS coatings and UV-PDMS/MCs/Ag coatings were tested by ZQ-90LA-5 electric tensile tester (Shanghai Qingji Instrumentation Technology Co., Ltd., Shanghai, China) at a compressive shear rate of 500 mm min^−1^ and a tensile rate of 20 mm min^−1^. The electrochemical properties of the UV-PDMS coatings and UV-PDMS/MCs/Ag coatings were recorded on PARSTAT 3000A electrochemical workstation (Ametek, Berwyn, PA, USA) using techniques such as electrochemical impedance spectra (EIS spectra), Bode spectra and potentiodynamic polarization curves (Tafel curves). The bacteriostatic properties of the UV-PDMS/MCs/Ag coatings were tested by colony counting, and the bacterial inhibition rate was calculated through electronic supplementary material, equation S1.

## 3. Results and discussion

### 3.1. Morphology of MCs

**Figure 2.**
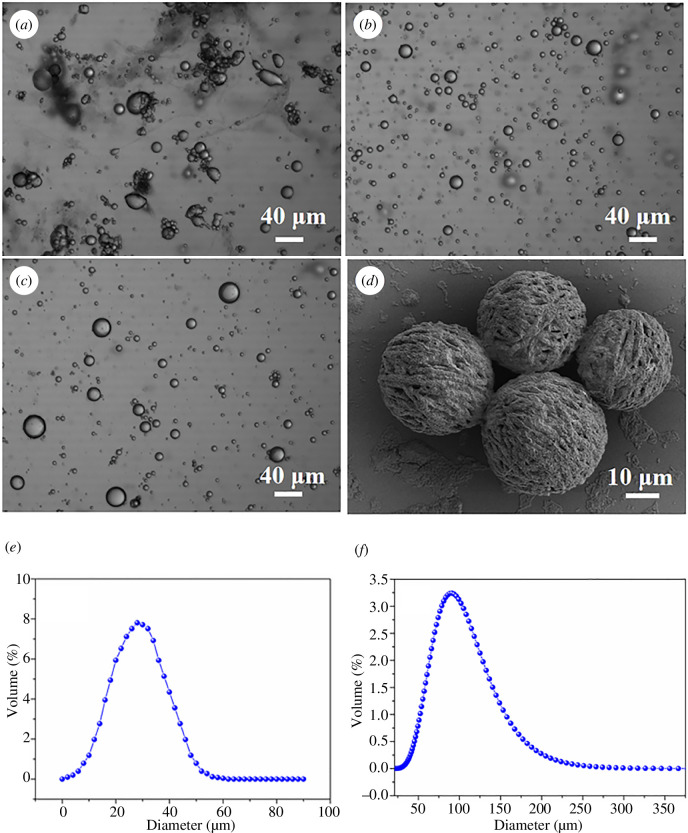
OM images of (*a*) before pH adjustment, and MCs formed under different reaction durations of (*b*) 1 h and (*c*) 4 h, and (*d*) SEM images of MCs. Size distribution of (*e*) MCs and (*f*) AgNPs.

The OM and scanning electron microscope (SEM) images of the MCs before pH adjustment and the MCs formed under different reaction durations are compared in figure 2. Many oval MCs were visible (figure 2*a*), issued from the reaction of ALG with CaCl_2_ to produce calcium alginate gel with a more serious agglomeration phenomenon. The resulting MCs shell was then assembled (figure 2*b*) to yield better MC monodispersity. During the process, the amino group of CS was protonated by hydrogen ions in a dilute acid solution to dissolve CS. The positive charge of CS with polycationic property led to the formation of a shell by electrostatic interaction with the carboxyl group of ALG [35]. Meanwhile, the surface became enriched by PGMA particles. After 4 h of microencapsulation reaction, the monodispersity of the MCs improved and the MCs gained better sphericity (figure 2*c*), indicating completed electrostatic adsorption of ALG and CS, as well as enrichment of PGMA particles on the surface of the shell layer. As displayed in figure 2*d*, the surface of MCs looked uneven owing to the formation of the shell layer of MCs by ALG and CS through the electrostatic action of the multi-layer polyelectrolyte membrane wraps. The size distribution of MCs and AgNPs was tested by DLS; the results were as shown in figure 2*e,f*. The average particle size of MCs and AgNPs was 30 μm and 90 nm, respectively, and these two different size scale particles were used to construct micro–nano dual-scale biomimetic structures.

In addition, the surfaces of the MCs were wrapped by numerous particulate matter formed by PGMA particles (electronic supplementary material, figure S1). The presence of PGMA particles on the shell layer would improve the stability and densification of the core wrapping, as well as extend the release cycle of the core materials. Overall, novel MCs were successfully prepared with the structure of the shell layer of the MCs consistent with what was hypothesized.

### 3.2. Structure of MCs

**Figure 3.**
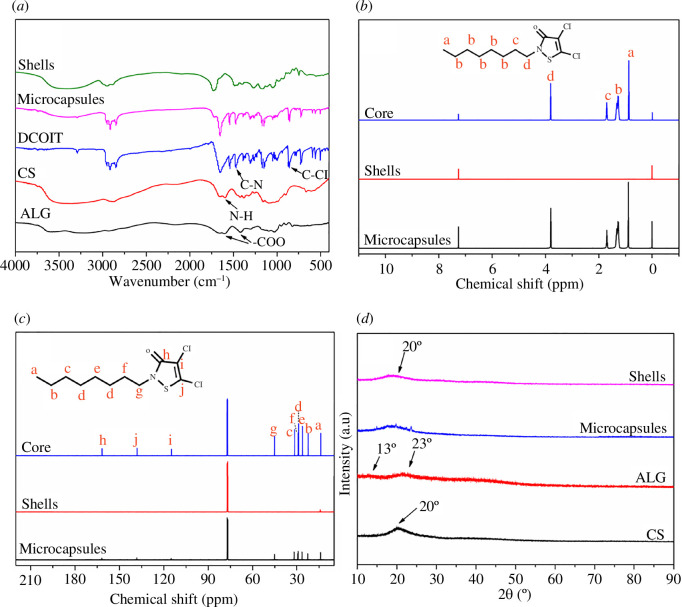
(*a*) FT-IR spectra of ALG, CS, DCOIT, shells and MCs, (*b*) ^1^H NMR spectra and (*c*) ^13^C NMR spectra of DCOIT, shells, MCs and (*d*) XRD spectra of ALG, CS, shells, MCs.

The FT-IR spectra of ALG, CS, DCOIT, shells and MCs are provided in figure 3*a*. The characteristic absorption peaks of ALG were located at 1591 cm^−1^ (–COO symmetric stretching vibration) and 1414 cm^−1^ (–COO asymmetric stretching vibration), while the characteristic absorption peak of CS was observed at 1595 cm^−1^ (N–H tensile vibration) [36]. The spectra of the MCs and shells revealed a broad peak at 3370 cm^−1^, which may be attributed to the formation of hydrogen bonding between ALG and CS. The disappearance of the N–H peak at 1595 cm^−1^ in MCs may be related to the inter-ionic interactions between –COOH and –NH_2_. Thus, the carboxyl group in ALG and the amino group in CS underwent electrostatic interactions to form a complex polyelectrolysis [21]. On the other hand, DCOIT displayed stretching vibrational absorption peaks of C–N bonds in the amide bonds near 1470 cm^−1^, as well as the stretching vibrational absorption peak of C–Cl bond at 855 cm^−1^ [37]. By comparison, the shells did not show peaks at these positions, whereas the MCs illustrated peaks. Therefore, DCOIT was successfully encapsulated by the MCs.

The ^1^H NMR spectra of MCs, shells and core are compared in figure 3*b*. The chemical shifts at 0.85–0.89 ppm corresponded to the H-a proton of the methyl fragment, while those at 1.21–1.37 ppm were assigned to the H-b proton. By comparison, the chemical shifts of the methylene protons H-c and H-d appeared at 1.70 and 3.80 ppm, respectively [38]. The spectrum of MCs revealed the presence of chemical shifts of DCOIT, suggesting a physical encapsulation process of the MCs. Therefore, DCOIT was successfully encapsulated by the MCs.

The ^13^C NMR spectra of MCs, shells and core are displayed in figure 3*c*. DCOIT showed octyl substituent chemical shifts at 14.04 ppm (C-a), 22.60 ppm (C-b), 26.40 ppm (C-e), 29.04 ppm (C-d), 29.34 ppm (C-f), 31.70 ppm (C-c) and 45.17 ppm (C-g) [38]. The carbon atoms of the five-membered heterocyclic ring resonated at 115.08 ppm (C-i), 138.21 ppm (C-j) and 161.85 ppm (C-h). By comparison, MCs presented all the signal peaks of DCOIT, indicating a physical encapsulation process of MCs. Thus, DCOIT was successfully encapsulated by the MCs.

The XRD spectra of ALG, CS, shells and MCs are provided in figure 3*d*. The block polymer aspect of ALG resulted in no obvious crystal diffraction peaks of crystalline materials, with characteristic peaks located at 2θ = 13° and 2θ = 23°. On the other hand, the characteristic diffraction peak of CS appeared at 2θ = 20° [39]. By comparison, the spectral features of the shells displayed declined peak intensities of both CS and ALG. Besides, the broadening of the diffraction peaks resulted in hidden diffraction peaks of ALG. Therefore, ALG and CS were attracted to each other through positive and negative charges, resulting in a reduced original degree of crystallization. As for the MCs, sharp diffraction peaks appeared at 2θ = 13°, 20° and 23°, which may be attributed to the solidification of DCOIT encapsulated in the MCs at lower temperatures, leading to an increased degree of crystallization. Also, the peak locations coincided with those of ALG and CS. Overall, the XRD results corroborated the FT-IR data, further confirming the formation of polyelectrolyte complexes by electrostatic attraction of the shells, as well as the successful preparation of MC.

### 3.3. Slow-release properties of MCs

**Figure 4.**
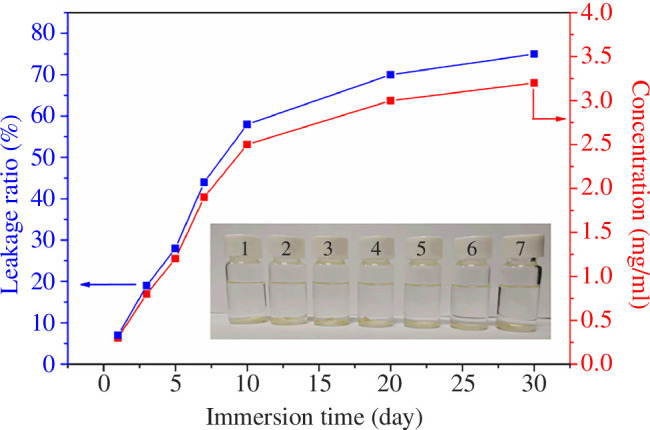
DCOIT release rates and release concentrations of MCs in xylene after different days (1–7 represents 1, 3, 5, 7, 10, 20 and 30 days, respectively).

As shown in electronic supplementary material, figure S2, the UV standard curve of DCOIT in xylene solution can be fitted with the equation: *y* = 1.3422*x −* 0.0126. Accordingly, the relationship between the absorbance and concentration can be written as: *A* = 1.3422*C* − 0.0126, where *A* represents the absorbance and *C* is the concentration. The DCOIT release rates and release concentrations of MCs in xylene after 1, 3, 5, 7, 10, 20 and 30 days are summarized in figure 4. At the early stage of release (1–7 days of immersion), the release rate of DCOIT was relatively fast due to the driving force provided by the large concentration difference between the inside and outside of the MCs for the release of DCOIT. However, the release rate of DCOIT gradually slowed down at the late stage of release, with a decrease in concentration difference between the inside and outside of the MC. After 7 consecutive days of release in xylene solvent, the release rate of MCs was only 44%. Since MCs may have a longer release cycle under mild seawater conditions, MCs with slow-release properties may ensure a long release cycle of the core.

### 3.4. Mechanical properties of UV-PDMS/MCs/Ag coatings

**Figure 5.**
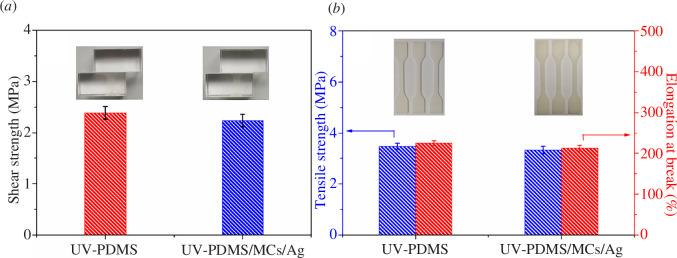
(*a*) Shear strengths of UV-PDMS coatings and UV-PDMS/MCs/Ag coatings, (*b*) tensile strengths and elongations at break of UV-PDMS coatings and UV-PDMS/MCs/Ag coatings.

The shear strengths of UV-PDMS coatings and UV-PDMS/MCs/Ag coatings are presented in figure 5*a*. The shear strength of UV-PDMS coatings on the glass substrate was estimated to be 2.39 MPa, and the shear strength of the UV-PDMS/MCs/Ag coatings on the glass substrate was 2.24 MPa. Hence, the addition of MCs and AgNPs reduced the shear strengths of the coatings, mainly due to the reduced adhesion area of the coatings and substrate by the added MCs. The mechanical properties of UV-PDMS coatings and UV-PDMS/MCs/Ag coatings are provided in figure 5*b*. The tensile strength of the UV-PDMS coatings was estimated to be 3.47 MPa and the elongation at break was 225%. After filling with MCs and AgNPs, the tensile strength and elongation at the break of the coating both declined to about 3.32 MPa and 212%, respectively. This decrease in tensile strength and elongation at break after filling with the MCs and AgNPs may be attributed to the addition of MCs, which reduced the cross-linking density inside the matrix, thereby the toughness and strength of the coatings. In a word, although the mechanical properties of UV-PDMS/MCs/Ag coatings are reduced after the addition of MCs and AgNPs, they still have excellent mechanical properties. In addition, the results of the friction resistance test are summarized in electronic supplementary material, figure S3. The coating contact angle is still greater than 150° after 500 friction tests, which shows that the coating has excellent abrasion resistance.

### 3.5. Antifouling properties of UV-PDMS/MCs/Ag coatings

**Figure 6.**
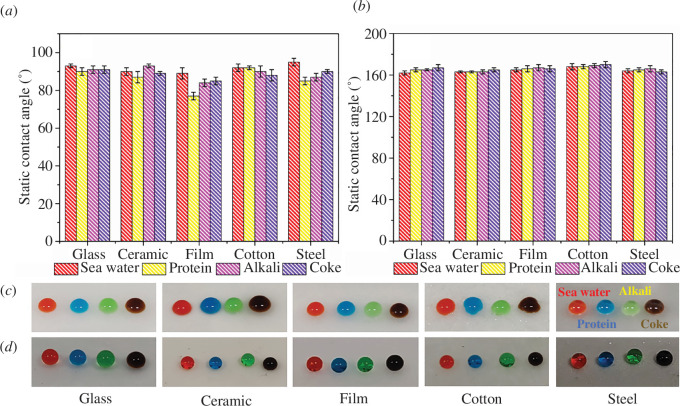
Static contact angles of different contaminants on (*a*) UV-PDMS coatings and (*b*) UV-PDMS/MCs/Ag coatings, photos of different contaminants on (*c*) UV-PDMS coatings and (*d*) UV-PDMS/MCs/Ag coatings.

The static contact angles of different contaminants on UV-PDMS coatings are gathered in figure 6*a*, and the static contact angles of different contaminants on UV-PDMS/MCs/Ag coatings are displayed in figure 6*b*. The static contact angle of UV-PDMS coatings was estimated to be about 90°, with poor antifouling effect. By comparison, the static contact angle of UV-PDMS/MCs/Ag coatings was above 160°. Thus, the addition of the MCs and AgNPs into the coatings greatly improved their antifouling performances due to the constructed micro–nano dual-scale structure on the surface of coatings by MCs and AgNPs [40].

### 3.6. Electrochemical properties of UV-PDMS/MCs/Ag coatings

**Figure 7.**
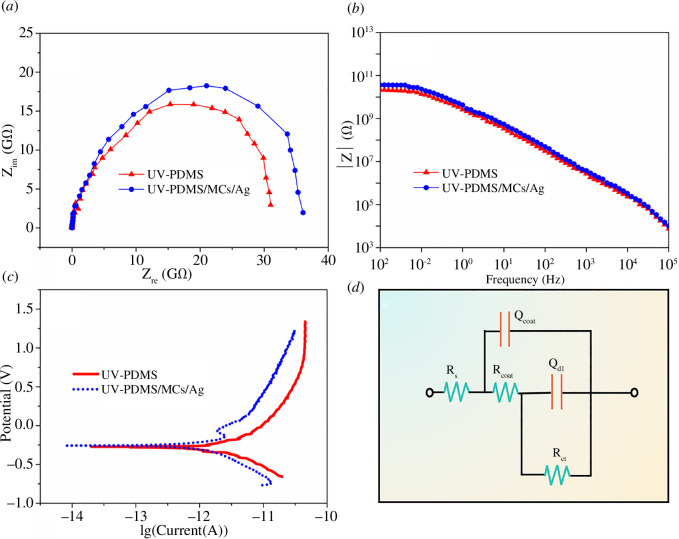
(*a*) Nyquist plots, (*b*) Bode plots, (*c*) Tafel plots of UV-PDMS coatings and UV-PDMS/MCs/Ag coatings and (*d*) equivalent circuit diagram.

The Nyquist plots of the UV-PDMS coatings and UV-PDMS/MCs/Ag coatings are presented in figure 7*a*. The impedance values of the coatings were all above 15 GΩ, suggesting better corrosion resistance. The Bode plots of UV-PDMS coatings and UV-PDMS/MCs/Ag coatings are depicted in figure 7*b*. In the low-frequency region, the |Z| values of UV-PDMS/MCs/Ag coatings were higher than those of UV-PDMS coatings. Hence, the corrosion resistance of UV-PDMS/MCs/Ag coatings was stronger than that of UV-PDMS coatings. Within the frequency range of 10^1^–10^5^ Hz, the slopes of both coatings were close to −1. At this time, the slopes of |*Z*| values can be linked to the electrochemical response properties of the coating capacitance. In other words, closer slopes of |*Z*| values to −1 should induce coatings closer to pure capacitors [41], suggesting corrosion resistance of both coatings suitable for reducing the substrate corrosion rate in the electrolyte [42]. The Tafel plots of UV-PDMS coatings and UV-PDMS/MCs/Ag coatings are shown in figure 7*c*. Compared with UV-PDMS coatings, the UV-PDMS/MCs/Ag coatings exhibited a higher ***U***_corr_ value and lower ***I***_corr_ value (electronic supplementary material, table S2). The results indicated that the filling of micro–nano particles had a spatial barrier effect in the coating and effectively slowed down the penetration of electrolyte. Meanwhile, the saline water immersion test and salt spray test also indicate that the coating has excellent corrosion resistance. The photos of saline water immersion test and salt spray test are shown in electronic supplementary material, figures S45, respectively. In addition, the equivalent circuit diagram model of UV-PDMS coatings and UV-PDMS/MCs/Ag coatings is displayed in figure 7*d*.

### 3.7. Antibacterial properties of UV-PDMS/MCs/Ag coatings

**Figure 8.**
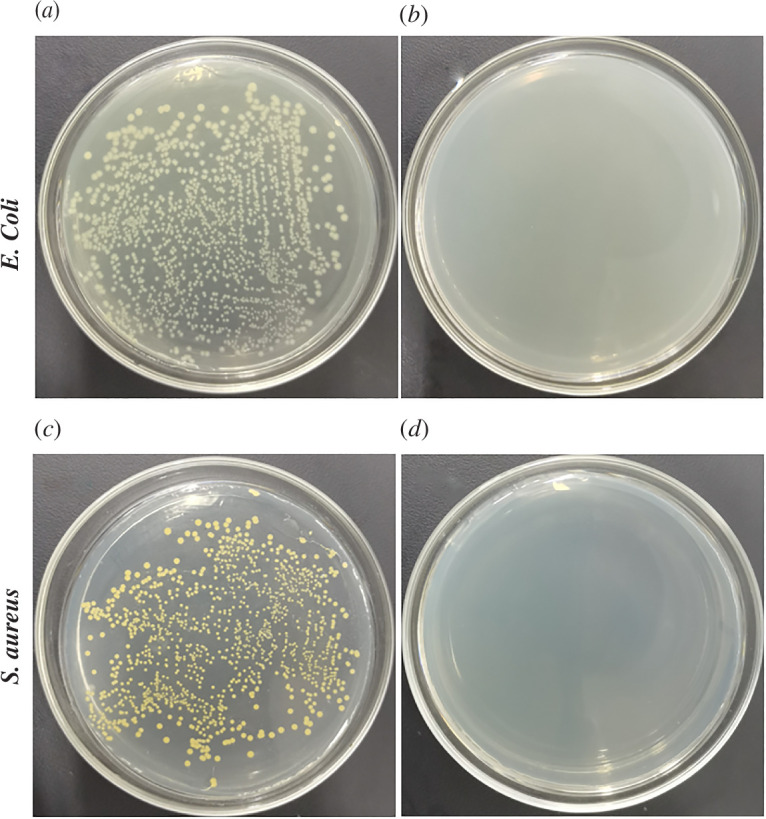
(*a*) *Escherichia coli* bacteria solution, (*b*) 200 μg ml^−1^ UV-PDMS/MCs/Ag coatings mixed with *E. coli* bacteria solution for 10 min, (*c*) *S. aureus* bacteria solution and (*d*) 200 μg ml^−1^ colony counting test photo of UV-PDMS/MCs/Ag coatings mixed with *S. aureus* bacteria solution for 10 min.

The experimental photos of the antibacterial rates of UV-PDMS/MCs/Ag coatings against *Escherichia coli* and *Staphylococcus aureus* are displayed in figure 8. Using UV-PDMS/MCs/Ag coatings, the bacterial inhibition rate of both *E. coli* and *S. aureus* at 200 μg ml^−1^ reached 100% within 10 min. Thus, UV-PDMS/MCs/Ag coatings possessed good bacterial inhibitory performances due to the slow release of DCOIT by MCs. Meanwhile, Ag ions were released from AgNPs in the UV-PDMS/MCs/Ag coatings, synergistically killing the bacteria. The excellent bacterial inhibition reduced the enrichment of bacteria on the surface of the coating and prevented the formation of biofilm on the coating surface [43], suitable for inhibiting the formation of large biomes.

## 4. Conclusion

MCs were successfully prepared by composite gel method using DCOIT as the core material and both ALG and CS as the shells. The obtained MCs showed slow-release performance with a release rate reaching only 44% for 7 days in the solvent. The combination of UV-PDMS coatings, MCs and AgNPs formed a micro–nano dual-scale biomimetic surface with environmentally friendly, long-term and stable antifouling properties. The as-obtained UV-PDMS/MCs/Ag coatings exhibited a static contact angle of about 160°, shear strength of 2.24 MPa, tensile strength of 3.32 MPa and elongation at a break of 212%. The DCOIT and AgNPs showed synergistic bacteriostatic effects, with the bactericidal rate of 200 μg ml^−1^ of UV-PDMS/MCs/Ag coatings towards *E. coli* and *S. aureus* reaching 100% within 10 min. In sum, the composite coatings illustrated good properties in terms of excellent mechanical, antifouling, bacteriostatic and long service life, making them promising for future use in marine transportation, pipeline networks and undersea facilities.

## Data Availability

The data have been uploaded in Dryad [44]. The data are also provided in the electronic supplementary material [45].
